# Aphrodisiac potentials of the ethanol extract of *Aloe barbadensis* Mill. root in male Wistar rats

**DOI:** 10.1186/s12906-017-1866-1

**Published:** 2017-07-11

**Authors:** Joseph O. Erhabor, MacDonald Idu

**Affiliations:** 1 Permanent Address: Phytomedicine Unit, Department of Plant Biology and Biotechnology, University of Benin, PMB 1154, Benin, Nigeria; 2 Phytomedicine Programme, Department of Paraclinical Sciences, Faculty of Veterinary Sciences, University of Pretoria, Pretoria, South Africa

**Keywords:** Aphrodisiac, Medicinal plant, *Aloe barbadensis*, Male sexual behaviour, Testosterone, Traditional medicine, Ethanol extract

## Abstract

**Background:**

*Aloe barbadensis* (AB) is a short stemmed succulent medicinal herb that is being used by locals in Nigeria to enhance libido. Therefore this study evaluates the aphrodisiac potential and acute toxicological effect of *A. barbadensis* (AB) root in male Wistar rats.

**Methods:**

Aphrodisiac potential was determined following the oral administration of graded doses (100, 200 and 400 mg/kg) of ethanol extract of *A. barbadensis* root. Sildenafil citrate (Viagra) and distilled water served as positive and negative controls respectively. Sexual behavioural parameters (mounting and intromission frequencies, mounting, intromission and ejaculatory latencies) were observed. Serum testosterone and cholesterol concentrations were also progressively monitored on days 1, 7 and 14. The acute toxicological evaluation of the plant were based on any onset behavioural changes and mortality respectively.

**Results:**

The findings from the sexual behavioural study indicated that the ethanol extract of *A. barbadensis* significantly increased mounting frequency and intromission frequency but significantly decreased mount and intromission latencies in a dose dependent manner particularly on day 1 and 14. The ethanol extract also prolonged ejaculatory latency. The testosterone and cholesterol concentrations were also increased as the dose increased particularly on day 1 and 7. The lowest dose of 100 mg/kg showed the best aphrodisiac effect. The toxicity studies showed that there were no acute behavioural changes with zero mortality.

**Conclusion:**

The increased blood testosterone and cholesterol concentrations by the ethanol extract of *A. barbadensis* can probably be said to be the possible mechanisms of action for its aphrodisiac property. The plant may also be used to treat hypotestosteronemia following its ability to increase testosterone. These findings therefore give backing to the acclaimed local use of *A. barbadensis* root as an aphrodisiac in males.

## Background

Over the years, medicinal plants have been used to manage an array of diseases/ailments. Nearly 80% of the populations of the world still rely on local medicines and traditional treatments mainly from plant extracts [1, 2]. It is noteworthy that herbal medicine is becoming very popular in the developing countries [3]. Today, the Nigerian traditional medicines are administered to treat a myriad of health problems including mental disorders, insomnia, broken bones and infertility as well as other reproductive health challenges [4].

It has been acclaimed that aphrodisiacs with a healthy lifestyle can achieve a better sexual life [5]. Sexual feelings are an inevitable part of life. The sex is the most cherished, indispensable and an integral part of every individual and can be a cradle of pleasure and satisfaction. There has been erroneous information, unawareness, fear and pessimistic outlook as far as sex is concerned. Myths and misconceptions are widespread and are passed on from one generation to another. These sexual myths can results in sexual dysfunctions, misery, silent suffering, distressed interpersonal relationships and even divorce. Sexual ignorance is a social disease and can be solved via compulsory all-inclusive sex education. This can boost awareness and improve the society [6].

However, aphrodisiac have been implicated in treating/managing these arrays of sexual disorders [6–9]. Most aphrodisiacs can be said to amplify some facets of sensual experience such as light, touch, smell, taste and hearing. This improved sensual consciousness leads to sexual stimulation and inclination [10]. Many locally accepted constituents have been known to increase libido in some continents of the world. Notable examples include Yohimbine, the Mandrake plant, ground Rhinoceros horn in the Chinese culture and the lethal “Spanish fly”. An aphrodisiac is any substance or agent (food, drug, scent or device) that stimulates the erotic instinct, induces veneral desire and surges pleasure and performance [8, 11, 12]. They are agents that can change impaired sexual functions [7]. Aphrodisiac substances can nevertheless be screened for activity by employing two methods: the observatory or physical method that encompasses the mating behavioural study (mount frequency and latency, intromission frequency and latency, ejaculatory latency and frequency, test for libido etc.) and biochemical method (hormonal determination, organ weight, histopathology, sperm count, motility and morphology etc). In either of the method, in vivo and in vitro animal models are used to ascertain aphrodisiac activity in laboratory animals such as rats, mice and guinea pigs [13]. Several reports have also attested to the use of animals particularly rats in assessing aphrodisiac activity of medicinal plants as found in [6, 7, 14–20].

Although, through history, a myriad of feature has qualified diverse substances as having aphrodisiac property, but are done generally via two probable approaches- cultural and scientific. Culturally, via the famous doctrine of signature, many plants and animals parts have been tented as having aphrodisiac effect. Popular among these, is the accepted belief of hunters of those eras when they consume definite parts of their prey to get the characteristics of those organs. In some part of the world (England and Ukraine), it is believed that plants with any Phallic-like characteristics such as asparagus, parsnips, celery and carrots were expected to have aphrodisiac property or effect. From our observation and as have been noted elsewhere, e.g. Rosen and Ashton [21], the root of AB is phallic-like in nature and culturally can be said to have an aphrodisiac effect. Scientifically, based on mechanism of action, aphrodisiacs can be classified into three categories- (aphrodisiacs that offer a high level of nutritional value, those with specific physiological effect and those that are psychologically active in nature. Following the phytochemical and nutritional constituents described elsewhere ([22], unpublished observation (Erhabor and Idu)), the extract of AB is said to have glycosides, flavonoid, phenol, tannin, alkaloids, steroids, reducing sugar, nitrogen free extract, crude fibre, crude protein, crude oil, ash content and moisture. Other nutritional components included sodium, potassium, magnesium, iron and zinc. Some of these phytoconstituents have been implicated for aphrodisiac activity in some plants (e.g. potassium, calcium, zinc and magnesium are known to be involved in processes that promote penile erection as reported by Jeon [23], Ghofrani [24], Adrogue and Madias [25], Coleman [26]. Alkaloids according to Zamblé et al. [27] dilate blood vessel which physiologically results to increase in blood flow to the penile organ and engorging of the penis for sexual performance. Phenols found in clove (*Syzygium aromaticum*) as observed by Tajuddin et al. [28] was said to be responsible for the aphrodisiac property of the plant. Also increase in testicular and/or serum cholesterol levels have been shown to be linked to the aphrodisiac activity of a medicinal plant. This is sequel to cholesterol being the precursor for the production of several physiologically important steroids that include bile acids, steroid hormones and vitamin D [7, 29, 30]. Following, an increase in cholesterol, it may lead to increased testosterone concentration via steroidogenesis which should normally reflect in a corresponding increase in libido [7].

Again, Local herbs have resulted in new breakthroughs in the treatment of sexual dysfunction and have become recognized worldwide as an immediate therapy [31]. Using plants as aphrodisiac in treating sexual disorders is gaining ground every day. Many of the effective herbal aphrodisiacs are accessible and have slight or no side effects [32]. Although, there are conventional treatment options but they have limited efficacy, hostile side effects and contraindications in some disease states [11]. The side effects of sildenafil citrate (Viagra)-a popular aphrodisiac drug include rashes, hypotension, facial flushing and urinary tract infection. Other side effects include back pain, nasal congestion, blurred vision, stomach upset, suicidal tendencies, mental disorders and dilation of the blood vessels [33, 34]. Other management therapies include surgery, psychiatric therapy, vacuum devices, penile implants and very expensive drugs which may not be affordable [35].

*Aloe barbadensis* (AB) is a short stemmed succulent herb in the Asphodelaceae family that have been acclaimed by locals in Nigeria to have libido enhancing property [36]. It has been refer to as being used in herbal preparations since the beginning of the first century AD. The extracts are said to have a rejuvenating, healing and soothing property as marketed in the cosmetic and alternative medicine industries [37, 38]. The leaf juice is used to treat intestinal ulcer and gynaecological problems and to treat catarrh [38]. It can be passed over flame and used to clear skin irritation such as ringworm and eczema. The root of *A. barbadensis* is used to cure constipation and impotency [39]. The fresh leaf juice is taken orally to treat stomach ulcer and externally to heal wounds [40]. It is used as a purgative, appetite-stimulant, emmenogogue and for managing colds, piles, asthma, cough and jaundice in ayurvedic formulation [41]. Previous reports show that the extract of AB have been proven via in vivo and in vitro studies to have effects on reproductive functions. Oyewopo et al. [42] observed that the extract had profound effect on the testicular weight and semen parameters of Sprague-Dawley rats. Iwu [43] also reported that 60 mg/kg b.w. of AB powder potentiated the rate of fertility and litter size of rabbits. However, from unpublished data (Erhabor, Obarisiagbon and Gabriel) a significant dose dependence increase (100, 200, 400 and 800 mg/kg) in sperm motility and count without any deleterious effect on the morphology was noticed. In another study by Ahmadi et al. [44] the extract of AB showed ability to influence reproductive functions in animals.

It is against this background, that this research was targeted at probing the potential activity of the ethanol extract of *A. barbadensis* root on the sexual behavior of male rats in order to determine the possible mechanism of action of the already implicated bioactives. It was also to corroborate the ethnomedical use of the plant as an aphrodisiac and against other male reproductive dysfunctions.

## Methods

### Collection and authentication of plant material

The roots of *Aloe barbadensis* were collected from Okene town in Okene local government area of Kogi State, Nigeria. It was initially identified by the authors (MI and JOE) both from the Department of Plant Biology and Biotechnology, University of Benin, Benin City, and further authenticated by Mr. Ibhanesebhor, G. of the Herbarium Unit of the Obafemi Awolowo University, Ile-Ife, Nigeria, with voucher number IFE17004 were the plant was deposited. The plant was also deposited at the herbarium of the Department of Plant Biology and Biotechnology, University of Benin, Benin City with voucher number UPBHx0160.

### Preparation of extract

The fresh roots of *Aloe barbadensis* were detached from the whole uprooted plant, rinsed in water and spread on laboratory tables where they were dried under room temperature. The plant material were then transferred to an oven set at 40 °C for 5–10 min before being reduced to fine powder with the aid of a mechanical grinder. 2000 g of the powdered plant material was extracted with 5000 ml of ethanol using a soxhlet extractor. The extract was concentrated to dryness using a water bath (HH-S Water Bath; Searchtech Instruments) set at an average temperature of 50 °C. The percentage yield of the ethanolic extract was determined using the formular (% Yield = weight of extract/weight of powder sample × 100/1).

### Drugs, assay kits and other reagents

Estradiol benzoate and progesterone were purchased from Sigma-Aldrich from China and USA. Sildenafil citrate was obtained from a community pharmacy outlet in Benin City, Edo State. The testosterone assay kit was procured from Monobind Inc., USA while every other chemicals used were of analytical grade.

### Animal groupings and extract administration

Seventy five (75) male and thirty 30 non oestrus female Wistar rats of 140–270 g and 145–260 g body weights was used for this study. The animals were acquired from the animal house of the Department of Anatomy, Faculty of Basic Medical Sciences, University of Benin, Nigeria. The animals were kept in sanitary wooden cages placed in well aerated animal house of the Department of Animal and Environmental Biology, Faculty of Life Sciences, for acclimatization with peak conditions (temperature, 25 °C; photoperiod, 12 h of natural light and 12 h of dark). The animals were permitted free access to water and fed with normal commercial pellets. The cages were cleaned daily throughout the period of the work. The animals were monitored once daily for their general health and weighed weekly. No adverse events was noticed before and during the experiment. The 75 male rats were wholly randomized into five groups of 15 and given appropriate treatment orally.

Group A was given the diluent (2 ml of distilled water) while group B, C and D was given 100, 200 and 400 mg/kg body weight, respectively, of *Aloe barbadensis* root extract. Group E was given the standard drug-Sildenafil citrate (5 mg/kg). The oral administration was carried out using orogastric tube. All animals used in this study were handled following the international guiding principles for biomedical research involving animals as outlined by the Council for International Organization of Medical Sciences and the International Council for Laboratory Animal Science [45].

### Mating Behavioural study

The mating behavioural tests was done following the modification of the procedures of [46–48]. From the groups (in a completely randomized manner), five male rats each were selected and observed for sexual behaviour after their diurnal doses on day 1, 7 and 14. The test was done between 19.00 and 03.00 h under a faint light in the Laboratory. The female animals were artificially brought into oestrus (heat) by the successive administration of estradiol benzoate (10 μg/100 g body weight) and progesterone (0.5 mg/100 g body weight) through subcutaneous injections, 48 h and 4 h respectively prior to pairing. This was done because the female rats only allow mating during the oestrus phase. The receptive female rats were introduced to the male rats, 30 min after administration of the extract at the respective doses to the male rats in a locally manufactured wooden cage with glass doors. The female rats were paired with the male rats in all the various doses including controls in the ratio 1:1 (1 female to 1 male). The observation for mating behaviour commenced after 10 min of placing the paired animals in the cage and was recorded with the aid of a video camera on a tripod stand. The test was discontinued if the male fail to manifest sexual interest. Any female animal that do not show receptivity was replaced by another artificially ‘heated’ female.

The occurrence of events and phases of mating after the video recording were analyzed and the frequencies and phases determined. The parameters of male sexual behavior as defined by [49] that were monitored after 35 min observation period includes: “Mount (MF) and Intromission frequency (IF) - the number of mounts and intromissions from the time of introduction of the female until ejaculation), Mount (ML) and Intromission latency (IL) - the time interval between the introduction of the female and the first mount or intromission by the male and Ejaculation latency (EL) - the time interval between the first intromission and ejaculation”.

### Test for libido

The level of sexual desire of the male rats was assessed by the protocol outlined in [12]. The libido test was carried out using the mounting and intromission frequencies as well their latencies of the mating behavioural test during the 1st, 7th and 14th day.

### Serum preparation

The modified technique as defined by Yakubu et al. [7] was used in the serum preparation. Blood was collected 1¼ hours after giving the extract, the standard drug (Viagra) and distilled water on day 1, 7 and 14. Under chloroform anesthesia, with the aid of a sterile forcep and scissors the stomach was cut open to expose the internal organs. The blood was collected via cardiac puncture using a 5 ml syringe and needle per animal into the appropriately labeled clean lithium heparin (to collect plasma) and non-coagulant(plain) (to collect serum) sample bottles. The sample bottles were kept at a temperature between 23 and 25 °C for ten minutes to clot. The bottles were centrifuged at 3000 rpm for ten minutes using a laboratory centrifuge. The sera and plasma collected were later aspirated with pasteur pipettes into dry plain bottles and utilized within 12 h of preparation for the testosterone and cholesterol assays.

### Determination of serum testosterone

The serum testosterone concentrations was determined quantitatively using the Microplate Enzyme Immunoassay kit for total testosterone concentration in human serum as described in the manufacturer’s test procedure (Accu-Blind ELISA Microwells, Product code: 3725–300, Monobind Inc., USA).

### Determination of plasma cholesterol

The method of Röschlau et al. [50] was used to determine the levels of total cholesterol, using enzymatic kits from Randox Laboratories Limited (LOT NUMBER: 2264CH), United Kingdom.

### Acute toxicity study

Twenty-five (25) male rats were utilized in this research and extract given as stated in the mating behavioural study earlier. The animals were completely randomized into five groups of five rats each. In all the groups the animals were monitored for 2 h for any behavioural changes such as hyperactivity, sedation, salivation, diarrhea, accelerated breathing, tail posture and convulsions after administering the extract (at the respective doses-100 mg/kg, 200 mg/kg and 400 mg/kg), distilled water and standard drug (viagra) to the corresponding groups. The mortality or lethality was counted after 24 h and the Lethal Dose (LD_50_) was determined. All animals were further observed for up to 14 days for any delayed mortality.

### Data analysis

Data were presented as mean ± SEM of five replicates. One Way ANOVA was done to compare means of different groups as well as a Duncan multiple range test to analyse differences among different means and the interaction between the variables using SPSS 15.0 computer software package. Differences at *P* < 0.05 or *P* < 0.01 were considered statistically significant.

## Results

### Percentage yield of extract

Two thousand grams of the powdered root plant material yielded 124.65 g (6.23%) of the ethanol extract.

### Effect of *Aloe barbadensis* ethanolic root extract on mating behaviours

**Fig. 1:**
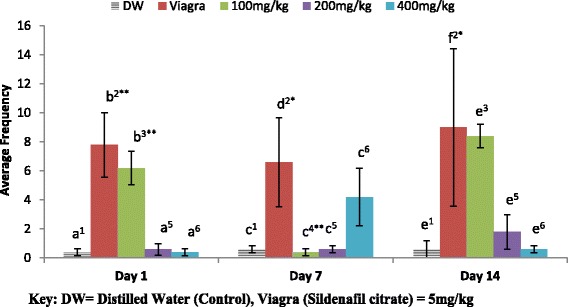
Effect of ethanol extract of *A. barbadensis* root on mount frequency of male rats. Bars with dissimilar alphabets are significantly different from each other on each day. Bars with different numbers for the same dose group at different days are significantly different. All values are expressed as Mean ± SEM; *n* = 5;**P < 0.01; *P < 0.05

The mounting frequency (MF) increased significantly on day 1 and 14 as the dose decreases as shown in Fig. 1. The effect of 100 mg/kg on MF was significantly different (*P < 0.05) from 200 and 400 mg/kg as well as the negative control (distilled water) except the positive control (Viagra) on day 1. On day 7 and 14, all dose groups were not significantly different from each other except the positive control (Viagra). Each of the dose groups on different days were not significantly different from each other except the 100 mg/kg group which was significantly different (**P < 0.01) on day 7 from the other days.

**Fig. 2:**
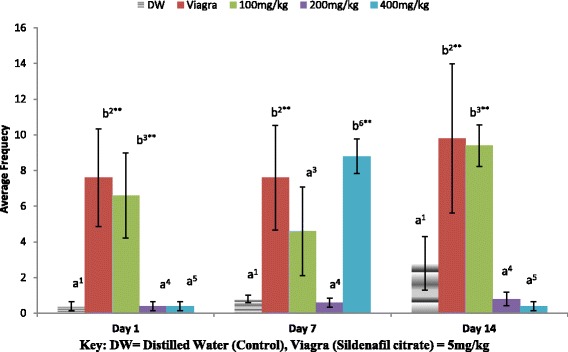
Effect of ethanol extract of *A. barbadensis* root on intromission frequency of male rats. Bars with unlike letters are significantly different from each other on each day. Bars with dissimilar numbers for the same dose group at different days are significantly different. All values are expressed as Mean ± SEM; *n* = 5;**P < 0.01; *P < 0.05

In Fig. 2, the 100 mg/kg of the extract exerted the greatest effect on Intromission frequency (IF) on day 1 and 14 as dose decrease while on day 7, 400 mg/kg had the highest effect. The 100 mg/kg and the positive control group (5 mg/kg of Viagra) were significantly different (**P < 0.01) from all other groups on day 1 and 14 while on day 7, it was the 400 mg/kg and the Viagra group that were significantly different (**P < 0.01) from the other dose groups. The highest dose group on day 7 was found significantly different (**P < 0.01) from the same dose groups on day 1 and 14.

**Fig. 3:**
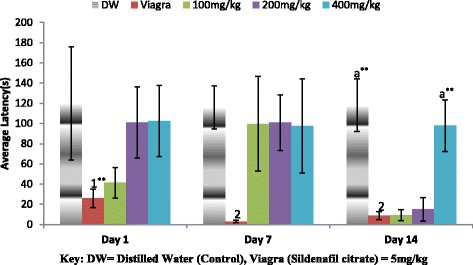
Effect of ethanol extract of *A. barbadensis* root on mount latency of male rats. Bars with dissimilar letters are significantly different; bars with letters are significantly different from bars without letters while those with no letters are not significantly different from each other on each day. Bars with different numbers for the same dose group at different days are significantly different while those without numbers are not significantly different. All values are expressed as Mean ± SEM; *n* = 5;**P < 0.01; *P < 0.05

Figure 3 shows the effect of the aqueous extract of *A. barbadensis* on mount latency. All dose groups on each of day 1 and 7 were observed not significantly different (*P < 0.05) from each other. The negative control (distilled water) and 400 mg/kg groups were found significantly different (**P < 0.01) from the other dose groups on day 14. However, there was an increase in mount latency (ML) on day 1 and 14 as the dose increases.

**Fig. 4:**
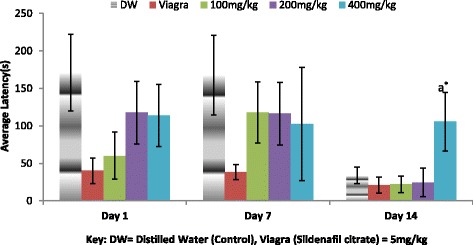
Effect of ethanol extract of *A. barbadensis* root on intromission latency of male rats. Bars with different letters are significantly different; bars with letters are significantly different from bars without letters while those with no letters are not significantly different from each other on each day. Bars with different numbers for the same dose group at different days are significantly different while those without numbers are not significantly different. All values are expressed as Mean ± SEM; *n* = 5;**P < 0.01; *P < 0.05

In Fig. 4 the effect of the aqueous extract of *A. barbadensis* on intromission latency was depicted. All dose groups on each of day 1 and 7 were observed not significantly different (*P < 0.05) from each other. The 400 mg/kg group was found significantly different (**P < 0.01) from the other dose groups on day 14. However, there was an increase in intromission latency (IL) on day 1 and 14 as the dose increases but the reverse was witnessed on day 7.

**Fig. 5:**
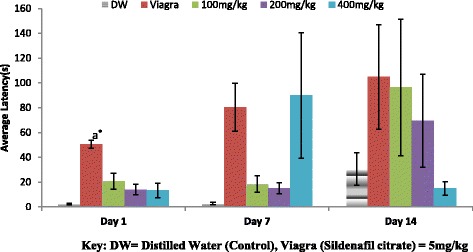
Effect of ethanol extract of *A. barbadensis* root on ejaculatory latency of male rats. Bars with different letters are significantly different; bars with letters are significantly different from bars without letters while those with no letters are not significantly different from each other on each day. Bars with different numbers for the same dose group at different days are significantly different while those without numbers are not significantly different. All values are expressed as Mean ± SEM; *n* = 5;**P < 0.01; *P < 0.05

The result of the effect of the aqueous extract of *A. barbadensis* on ejaculatory latency (EL) is shown in Fig. 5. It was observed that within the days and across the days at the various dose groups only the Viagra group on day 1 was found significantly different from the other dose groups. There was a decrease in ejaculatory latency on day 1 and 14 as the doses increase as against an increase observed on day 7.

### Effect of *Aloe barbadensis* ethanolic root extract on serum testosterone levels

**Fig. 6:**
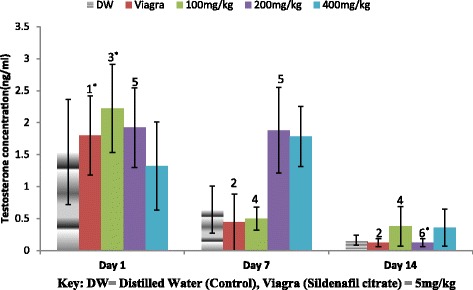
Effect ofethanol extract of *A. barbadensis* root on the serum testosterone concentration of male rats. Bars with unlike letters are significantly different; bars without letters are significantly different from bars with letters while those with no letters are not significantly different from each other on each day. Bars with different numbers for the same dose group at different days are significantly different while those without numbers are not significantly different. All values are expressed as Mean ± SEM; *n* = 5;**P < 0.01; *P < 0.05

The administration of the extract of *A. barbadensis* at 100 mg/kg significantly increased (*P < 0.05) serum testosterone concentration on day 1 when compared to day 7 and 14 (Fig. 6). The extract when given at 200 mg/kg lead to a significant decrease (*P < 0.05) on day 14 when compared to day 1 and 7. However a significant decrease (*P < 0.05) in testosterone concentration was observed in the Viagra group across the days (Fig. 6).

### Effect of *A. barbadensis* ethanolic root extract on cholesterol

**Fig. 7:**
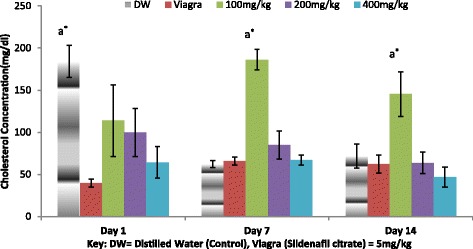
Effect of ethanol extract of *A. barbadensis* root on cholesterol concentration of male rats. Bars with unlike letters are significantly different; bars with letters are significantly different from bars without letters while those with no letters are not significantly different from each other on each day. Bars with different numbers for the same dose group at different days are significantly different while those without numbers are not significantly different. All values are expressed as Mean ± SEM; *n* = 5;**P < 0.01; *P < 0.05

The effect of the ethanol extract of *A. barbadensis* on cholesterol levels of the male rat are shown in Fig. 7. A gradual decrease in cholesterol concentrations was observed as the dose levels increases. On day 1, all treated groups and the positive control group (Viagra) were found not significantly different from each other but significantly different (*P* < 0.01) from the negative control (distilled water). The 100 mg/kg dose group was observed on day 7 and 14 to be significantly different from the other dose groups including controls.

### Acute toxicological study

Results from the acute toxicological studies revealed that the ethanol extract of *A. barbadensis* was safe up to the highest dose of 400 mg/kg. No toxic symptoms or adverse behavioural changes were observed as zero mortality was recorded during the period of the study.

## Discussions

### Percentage yield of extract

The percentage yield of the crude extract showed a high yield of 124.65 g (6.23%) from the 2000 g root powder when compared with *Persea americana* which yielded 95.55 g (4.78%) [51].This may be due to differences in solvent, as well as the extraction/concentration procedures used.

### Mating behavioural study

Using animal model for the initial screening to determine the aphrodisiac potential of a test drug is an accepted model. This mouse model is simple and quick [52] and can also be said to be used to evaluate the aphrodisiac and stimulating activity on penile erection against erectile dysfunction [53]. Sexual behavioural parameters such as mount and intromission frequencies are indices of sexual vigour, libido and potency [28, 54].

The mating behavioural test revealed that the ethanol extract of *A. barbadensis* root increased mount and intromission frequencies when compared with the negative control group, though the effect was less than that of viagra (Figs. 1 and 2). It also decreased the mount and intromission latencies in the male rats (Figs. 3 and 4) and prolonged the ejaculatory latencies (Fig. 5) on day 1, 7 and 14. These significant increases in mounting frequencies (MF) and intromission frequencies (IF) with corresponding decreases in mount latency (ML) and intromission latency (IL) are indications that the male rats were aroused. It also reflects enhanced performance, motivation and vigour. These findings agree with earlier report by Ratnasooriya and Dharmasiri [55]; Yakubu and Afolayan, [56]; Yakubu and Akanji [16]; Gbankoto et al. [18] on the significant changes in ML and IL. Also, the prolonged ejaculatory latency (EL) by the ethanol extract of *A.barbadensis* is a strong indication that the sexual function of the male rats was enhanced (prolonged duration of coitus) suggesting an aphrodisiac activity. These findings which is similar to the report by Fouche et al. [17] further support the activity of *A. barbadensis* root ethanol extract in enhancing sexual function. However, the highest dose of 400 mg/kg had reversed activity/inhibition on sexual behavioural parameters on day 1 and 14. This agreed with the findings of Ratnasooriya and Dharmasiri [55]; Yakubu and Afolayan [54] were they observed the same reverse inhibition at the highest dose of 3000 mg/kg of *Terminalia catappa* seeds and 100 mg/kg of *Bulbine natalensis* stem in their respective studies. This may be due to sedation as animals showed no form of sexual interest.

It has been earlier reported that androgens are important modulators of male sexual behaviour including erection and libido. These androgens may act both at the central and peripheral nervous system levels [57, 58]. Testosterone is one of the main androgens in the male gonads produce by the interstitial Leydig cells of the testis [49]. Testosterone administration had been reported to enhance sexual function and libido. It also, improved the intensity of orgasm and ejaculations [59, 60]. An increase in testosterone had been linked with a moderate but corresponding increase in sexual desire or libido [57, 59, 61]. It was observed that the administration of the extract at 100 mg/kg resulted in the highest testosterone concentration on day 1 and 14 while the 200 mg/kg of the extract on day 7, produced the highest concentration of testosterone level (Fig. 6). This may have accounted for the profound effect on sexual and masculine behavioural parameters of the male rats.

Reports suggest that cholesterol is a requirement for normal activity of the testicles. Cholesterol is also a known precursor in the synthesis of the steroids including bile acids, steroid hormones and vitamin D [29, 30]. Yakubu et al. [7, 12] reported that an increase in testicular and/or serum cholesterol concentrations led to a corresponding increase in the aphrodisiac activity of a medicinal plant. This increase in cholesterol concentrations results in increased production of testosterone which translates into increased libido. It was found that the extract increased cholesterol concentrations (Fig. 7) corroborating the increase testosterone concentrations observed.

### Toxicological evaluations

All animal in all the groups, showed no significant adverse acute toxicological effect that can be attributed to the acute administration of the ethanol extract of *A. barbadensis.* Also, adverse changes in behaviour were not observed, indicating that physical clinical signs were unremarkable. The intake of food and water were normal, suggesting that the animals had a normal appetite. No mortality was noticed during the entire period of the study. It can therefore be inferred that the lethal dose (LD_50_) of the extract is greater than 400 mg/kg since up to this dose no death was recorded. This findings agrees with previous report by Gatsing et al. [62] were 0 % mortality when the aqueous leaf extract of *Alchornea cordifolia* at 3200 mg/kg and the methanol and aqueous leaf extracts of *Emilia coccinea* at 8000 mg/kg were administered [63].

## Conclusions

Overall, this study showed that the ethanol extract of *A. barbadensis* root has aphrodisiac potential which had lend credence to its traditional use as an aphrodisiac agent in Nigerian traditional medicine. The lowest dose of 100 mg/kg of the extract presented the best aphrodisiac effect. The extract has a functional capacity to increased testosterone and cholesterol concentrations which are possible mechanisms of action for its aphrodisiac property. The ability of the plant to relax the corpus cavernosum muscle of the penile organ as another possible mechanism of action is being considered.

## Abbreviations

AB: *Aloe barbadensis*
DW: Distilled water
EL: Ejaculatory latency
IF: Intromission Frequency
IL: Intromission Latency
MF: Mounting Frequency
ML: Mounting Latency
